# Effect of Different Printing Designs and Resin Types on the Accuracy of Orthodontic Model

**DOI:** 10.3390/polym17202724

**Published:** 2025-10-10

**Authors:** Sabahattin Bor, Fırat Oğuz

**Affiliations:** Department of Orthodontics, Faculty of Dentistry, İnönü University, Malatya 44280, Türkiye; venaroshan@gmail.com

**Keywords:** 3D printing, accuracy, deviation analysis, color map visualization, in-house aligner fabrication, dental resins

## Abstract

This study aimed to evaluate the effect of resin type and printing design on the dimensional accuracy of three dimensional (3D) printed orthodontic models, considering their clinical relevance for applications such as in-house aligner fabrication. Since low-cost Liquid Crystal Display (LCD) printers have been increasingly adopted in practice but data on their trueness and precision with different resins and print designs were limited, the study sought to provide evidence-based insights into their reliability. A mandibular model was designed using Blenderfordental (B4D, version 1.1.2024; Dubai, United Arab Emirates) software and fabricated with the Anycubic Photon Mono 7 Pro 14K (Anycubic, Shenzhen, China) LCD printer. The model was printed in vertical orientation using three different print designs at two layer thicknesses (50 µm and 100 µm). Four resins (Elegoo, Anycubic, eSUN, and Phrozen) were used, and each resin was printed with all three designs, yielding 126 models per resin and a total of 504 printed models. Dimensional deviations between the printed and reference models were assessed using root mean square (RMS) values and color-coded deviation maps. Significant differences in trueness were found among resins and print designs at both layer thicknesses (*p* < 0.001). At a layer thickness of 50 µm, eSUN and Anycubic showed superior trueness, whereas Phrozen exhibited the highest deviations. At a layer thickness of 100 µm, Anycubic, eSUN, and Phrozen generally performed better than Elegoo. Overall, printing at 100 µm yielded better performance than at 50 µm. Precision analysis revealed resin-dependent differences, with eSUN showing significantly higher precision than Elegoo at both layer thicknesses (*p* = 0.006 at 100 µm, *p* < 0.001 at 50 µm) and superior precision compared to Phrozen at 50 µm (*p* = 0.019). Both resin selection and print design significantly affect the dimensional accuracy of 3D-printed dental models.

## 1. Introduction

Clear aligner therapy has emerged as an important aesthetic alternative in orthodontic practice, driven by the growing demand for orthodontic treatment among adult patients [1]. In addition to providing enhanced aesthetics, aligners improve patient comfort, facilitate oral hygiene maintenance, and impose fewer dietary restrictions. Studies have also reported higher levels of satisfaction and acceptance of aligners among adults compared with fixed orthodontic appliances [2,3].

The conventional digital workflow for producing clear aligners involves three dimensional (3D) printing digital models generated from orthodontic setups, followed by sequential alignment stages [4]. While this process was traditionally performed by aligner firms, the widespread adoption of 3D printing technologies has made in-office production increasingly feasible [5]. This advancement allows clinicians to fabricate custom orthodontic appliances, including clear aligners, directly within the clinical setting [4,6]. Consequently, clinicians now play a more active role in the design and production process, reducing dependence on aligner firms. In-office production offers distinct advantages, such as faster delivery of aligners and the ability to rapidly re-plan in the event of errors, thereby contributing to more efficient clinical workflows compared with reliance on aligner firms [6,7].

The application of computer-aided design (CAD) and computer-aided manufacturing (CAM) has become increasingly widespread in dentistry. The CAM process is often synonymous with subtractive manufacturing [8], in which the designed product is fabricated from a solid block through milling and polishing with specific tools. However, subtractive techniques such as milling can result in material loss of up to 90% [9]. Due to these disadvantages, additive manufacturing has emerged as a prominent alternative for producing dental appliances from CAD files.

Additive manufacturing processes are fundamentally based on the layer-by-layer construction of physical objects using three-dimensional design data created in a digital environment. This method allows for the high-precision production of objects with complex geometries by adding material in thin layers. The term “additive manufacturing” is also commonly referred to as “3D printing” [8]. Unlike subtractive methods, 3D printing can reduce material waste and enable the fabrication of more intricate geometries.

Today, the 3D printing process is widely used across various fields of dentistry. In prosthodontics, it is employed in the production of crowns, bridges, and removable dentures. In surgery, it is used for the fabrication of surgical guides, occlusal splints, and other guiding appliances [10]. In orthodontics, 3D printing is frequently utilized for manufacturing surgical guides for miniscrew placement, custom orthodontic appliances, bracket transfer trays, and clear aligners [11,12,13].

The dimensional accuracy of 3D-printed models is of critical importance in orthodontics, as appliances and clear aligners must fit accurately on the dental tissues. Even micron-level deviations may affect appliance adaptation and influence treatment outcomes. According to ISO 5725 standards [14], accuracy consists of two elements: trueness and precision. Trueness describes the closeness of agreement between the printed model and the actual dimensions of the digital design, while precision refers to the reproducibility of results when the printing process is repeated under the same conditions [15]. The clinical success of 3D-printed appliances depends on achieving a balance between these two aspects and controlling the factors that influence them during production. In orthodontics, accurate transfer of planned tooth movements from the digital setup to physical models ensures accurate realization of planned outcomes, potentially reducing the number of required aligners and improving both efficiency and predictability of treatment.

Numerous factors can influence the accuracy of 3D printed dental models, and these can be grouped into several categories. Printer type and polymerization technique play a primary role, as different technologies such as stereolithography (SLA), digital light processing (DLP), and liquid crystal display (LCD) vary in the way the liquid resin is cured within the vat system [4,16,17,18,19,20,21,22,23,24,25,26]. Previous evaluations comparing SLA, DLP, and LCD systems have shown that SLA printers generally achieve the highest accuracy, while entry level LCD printers tend to produce results closer to the threshold of acceptable accuracy. Nevertheless, these models typically remain within the clinically acceptable error limit of 0.25 mm, making LCD printers increasingly popular for their affordability and rapid production capabilities [4,15,16,19].

The arrangement of models on the printer platform also influences accuracy [19]. Some findings indicate that the front area of the build platform yields the most accurate prints, whereas others suggest that objects located in the center achieve higher accuracy than those positioned near the edges [24,25]. Regarding anatomical characteristics, studies examining models with and without dental crowding reported variations in accuracy [4].

In addition, the accuracy of 3D printing can vary significantly depending on the type of polymer-based resin used, with a strong positive correlation observed between lower volumetric shrinkage and higher dimensional accuracy [27]. However, a comprehensive review of the literature demonstrates that studies evaluating the effect of different resins on dimensional accuracy remain limited. Polymerization shrinkage, an inherent limitation of conventional (meth)acrylate-based chemistries, is a critical issue during photopolymerization that can result in poor accuracy, uneven surfaces, and even warpage and curling of the printed model [27,28,29]. This process can lead to the formation of polymer networks with low uniformity and high brittleness, which are less efficient at dissipating stress [29]. Consequently, achieving dimensionally stable and accurate models is a key step for clinical success in orthodontic applications, where precision directly impacts treatment outcomes [27].

Material properties such as light transmittance, viscosity, polymer network structure, and curing behavior directly influence the accuracy and structural integrity of the final product. Recent research has demonstrated that specific compositional elements, such as the ratio of cross-linking agents to base monomers, significantly affect both the printability and mechanical properties of the resulting models [28]. The viscosity of printing resins increases with higher polymer content, while mechanical properties improve with increased cross-linker concentration, creating a delicate balance that must be optimized for each application [28]. In this context, the utilization of advanced resins with low polymerization shrinkage, potentially employing alternative strategies like radical step-growth mechanisms, is highly desirable for obtaining reliable and clinically acceptable outcomes [27,29].

Furthermore, factors beyond material choice, such as the strategic addition or omission of support structures, the printing technology used, post-processing methods (including washing and post-curing protocols), and aging, have been identified as significant factors influencing dimensional accuracy [27,28]. The degree of conversion of carbon-carbon double bonds during polymerization also plays a crucial role in determining the final properties and dimensional stability of printed objects [28]. Additionally, the vertical layer thickness defined in slicing software remains a critical parameter that directly affects printing outcomes [4,30,31,32].

The aim of this study is to compare the dimensional accuracy of orthodontic models printed from different resin types and under conditions with and without support structures, using the same digital model. The alternative hypothesis was formulated as follows:

**H_1_:**  *Printing design, resin type, and layer thickness affect the dimensional accuracy of orthodontic models produced by 3D printing.*

## 2. Materials and Methods

### 2.1. Model Design and Planning

**Figure 1:**
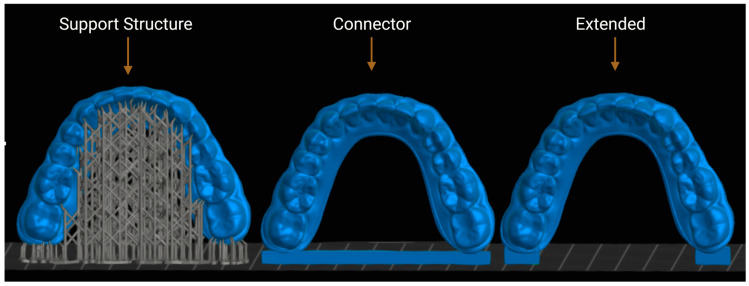
Three different 3D printing design approaches for same orthodontic model.

The mandibular model was designed using Blenderfordental (B4D, version 1.1.2024; Dubai, United Arab Emirates) software. Subsequently, planning was performed with three different print designs: with supports, without supports, and with connectors in the posterior lower region, using Chitubox Dental Slicer software v1.2.0 (Shenzhen, China) (Figure 1).

### 2.2. Three-Dimensional Printing Process

The printer used in our study was a recently released and affordable model, categorized among low-cost printers, the Anycubic Photon Mono 7 Pro 14K (Anycubic, Shenzhen, China), equipped with a high-resolution 14K LCD screen. In LCD-based 3D printing, release films such as FEP (fluorinated ethylene propylene), NFEP (commonly marketed as nFEP; often referring to PFA—perfluoroalkoxy), and ACP (Advanced Composite Film) are commonly used (https://helpcenter.phrozen3d.com/hc/en-us/articles/6399641736857-What-are-the-Differences-Between-FEP-PFA-nFEP-and-ACF-Films?, accessed on 31 August 2025). Among these, the latter two—PFA and ACP—have recently attracted attention in new-generation printers. The printer utilized in our study is equipped with ACP film, and it is believed that the technical properties of the release film can influence printing accuracy.

Four commercially available resins were selected for this study, including two orthodontic, one dental model, and one general-purpose resin. All models were printed vertically at 50 µm and 100 µm layer heights (Table 1).

The sample size of the study was calculated using the G*Power (version 3.1, Heinrich Heine University, Düsseldorf, Germany) software. The analysis was performed with the assumptions of an effect size of f = 0.25, α = 0.05, and a power of 0.80 [33]. Based on the study design, which included 4 different resins, 3 different printing options, and 2 different layer thicknesses, a total of 24 groups were formed. The calculation indicated that at least 19 samples per group, amounting to a total of 465 models, were required. In the present study, 21 models were obtained for each group, resulting in a total of 504 models, thereby exceeding the minimum required sample size and ensuring adequate statistical power.

### 2.3. Print Optimization

Before the main printing, the printer was calibrated in terms of the Z-axis for each resin. Additionally, 8 standard Resin Exposure Range Finder (R_E_R_F) test prints were made for each resin to determine optimal exposure settings individually. In the initial test prints, it was observed that support-free models did not adhere to the build platform. To overcome this issue, the bottom exposure time was increased. Additionally, the lift and retract speeds were reduced.

### 2.4. Post-Print Processing

In post-print processing, water was used to remove uncured resin from water-washable resin prints. For other resins, a two-stage cleaning process was performed using 99% isopropyl alcohol, with each stage lasting 3 min. For post-print curing, the Ackuretta Curie (Ackuretta Technologies, Taipei, Taiwan) machine was used for 10 min.

### 2.5. Accuracy Assessment

All printed models were scanned and digitized using laboratory-type SmartOptics scanner (Smart Optics Sensortechnik GmbH, Bochum, Germany). Two concepts were employed to assess accuracy: trueness, defined as the degree of deviation of the printed model from the reference model, and precision, defined as the consistency between repeated prints. For surface deviation analysis, two separate Blender-based tools were employed. The first was a commercially purchased Blender addon, into which the Open3D (version: 0.19) library was integrated to extend its capabilities [34].

**Figure 2:**
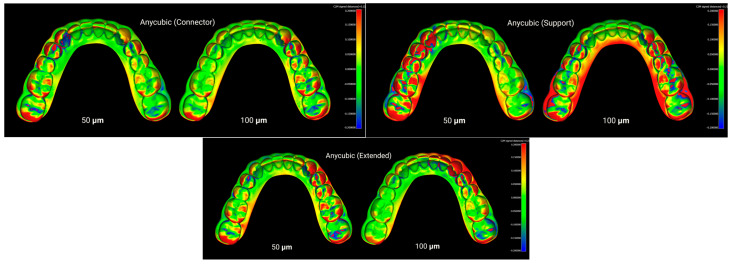
Superimposition of Anycubic resin models printed with connector, support, and extended designs at 50 µm and 100 µm layer thicknesses.

**Figure 3:**
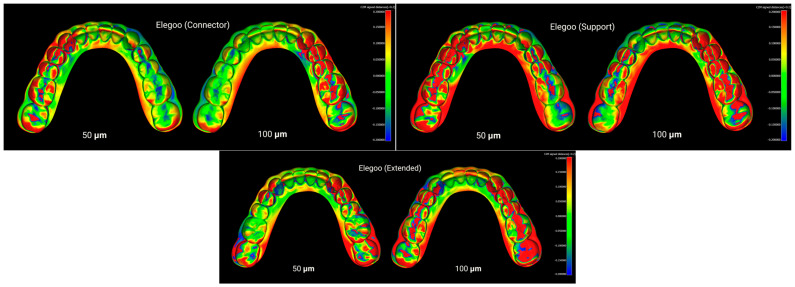
Superimposition of Elegoo resin models printed with connector, support, and extended designs at 50 µm and 100 µm layer thicknesses.

**Figure 4:**
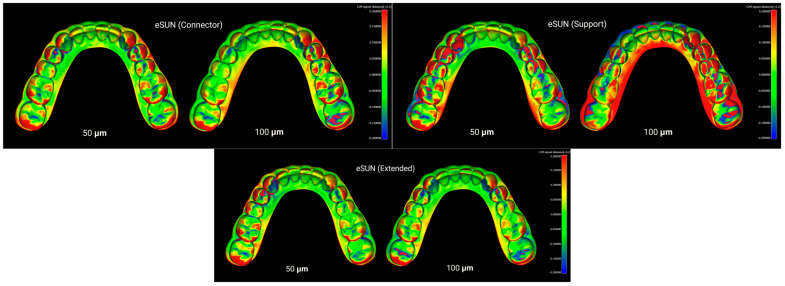
Superimposition of eSUN resin models printed with connector, support, and extended designs at 50 µm and 100 µm layer thicknesses.

**Figure 5:**
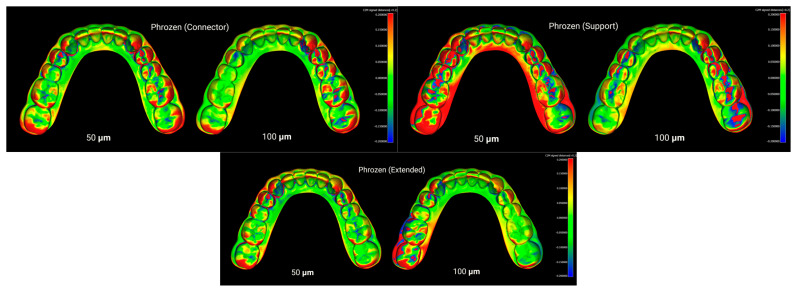
Superimposition of Phrozen resin models printed with connector, support, and extended designs at 50 µm and 100 µm layer thicknesses.

Furthermore, CloudCompare (version 2.13.2, Kharkiv) was employed as an additional advanced software for fine alignment and measurement. In this workflow, an initial basic alignment was performed in Blender using ICP. Unwanted regions of the models were then trimmed in Blender. Subsequently, the aligned reference and test STL files were imported into CloudCompare, where a final fine registration was carried out to achieve optimal superimposition. The RMS values obtained from this step were exported to an Excel file (Microsoft Excel, Office 365) for statistical analysis. As a result, four distinct groups were formed based on the type of resin used, and three groups were defined based on the print design: with supports, without supports, and with connectors in the posterior lower region (Figure 2, Figure 3, Figure 4 and Figure 5). To assess method reliability, the same researcher repeated the superimposition process for 20 models following the same workflow.

### 2.6. Statistical Analysis

Statistical analysis was performed using R language (4.5; R Foundation for Statistical Computing, Viena, Austria) and RStudio 2025 (Version 2025.05.1 + 513; Posit Software, PBC, Boston, MA, USA). Data were grouped based on resin type (four groups: Anycubic, Elegoo, eSUN, and Phrozen), layer thickness (two levels: 50 µm and 100 µm), and print design (three types: Support, Extended, and Connector).

Normality of the data distribution was assessed using the Shapiro–Wilk test. Homogeneity of variances was evaluated using Levene’s test. For normally distributed data with equal variances, one-way ANOVA was performed, followed by Tukey’s HSD post hoc test. When normality was satisfied but variances were unequal, Welch’s ANOVA was applied, followed by the Games–Howell post hoc test. If the normality assumption was not met, the Kruskal–Wallis test was applied, followed by Dunn’s test for post hoc analysis. Figures were generated using the ggplot2 package in R. The significance level was set at *p* < 0.05.

### 2.7. Method Error

To evaluate method error, the same operator re-aligned the identical models after a one-month interval, and root mean square (RMS) values were recorded as deviation measurements. The consistency between the first and second measurements was analyzed using the Intraclass Correlation Coefficient (ICC). The ICC was calculated as 0.92, indicating excellent repeatability of the measurements.

## 3. Results

### 3.1. Resin-Based Comparisons

**Figure 6:**
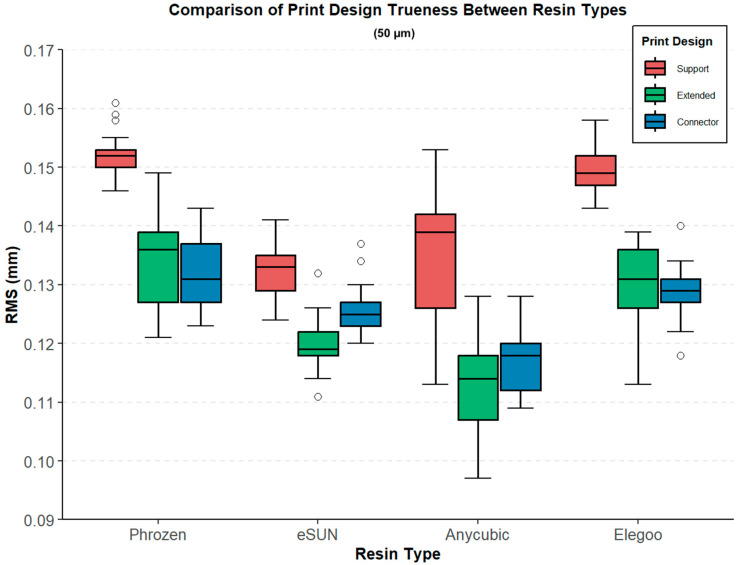
Comparison of print design trueness (RMS, mm) between different resin types (Phrozen, eSUN, Anycubic, Elegoo) at 50 µm layer thickness. Boxplots represent three print designs: support (red), extended (green), and connector (blue).

At 50 µm layer thickness, the RMS values showed significant differences among both design types and resin groups (Table 2, Figure 6). In the Support design, the lowest RMS value was recorded in the eSUN group (0.132 ± 0.004 mm), while the highest was observed in the Phrozen group (0.152 ± 0.004 mm) (Welch ANOVA, F(3, 43.3) = 106.003, *p* < 0.001). For the Extended design, the Anycubic group exhibited the lowest RMS value (0.114 ± 0.008 mm), whereas the Phrozen group showed the highest RMS value (0.135 ± 0.008 mm) (F(3, 42.6) = 32.350, *p* < 0.001). In the Connector design, RMS values ranged from 0.118 ± 0.006 mm (Anycubic) to 0.132 ± 0.006 mm (Phrozen), with statistically significant differences across groups (F(3, 44.0) = 20.708, *p* < 0.001).

**Figure 7:**
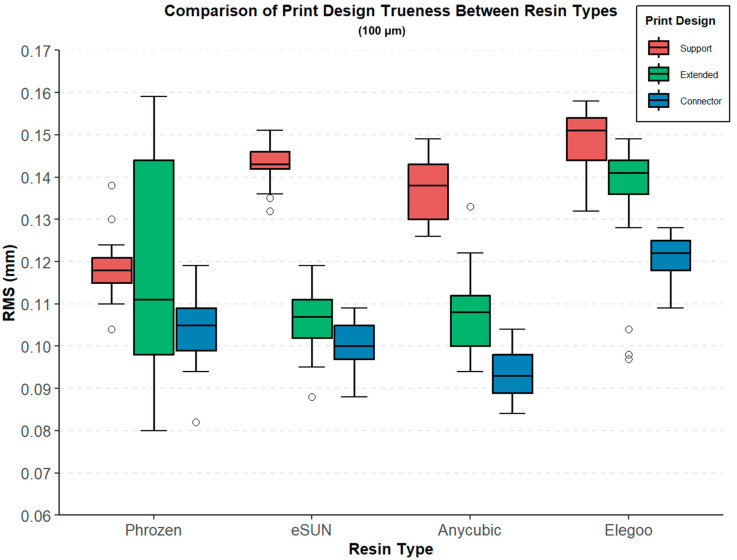
Comparison of print design trueness (RMS, mm) between different resin types (Phrozen, eSUN, Anycubic, Elegoo) at 100 µm layer thickness. Boxplots represent three print designs: support (red), extended (green), and connector (blue).

At 100 µm layer thickness, the RMS values demonstrated statistically significant differences among both design types and resin groups (Table 3, Figure 7). In the Support design, the lowest RMS value was observed in the Phrozen group (0.119 ± 0.007 mm), whereas the highest was found in the Elegoo group (0.149 ± 0.007 mm) (Welch ANOVA, F(3, 43.4) = 75.899, *p* < 0.001). For the Extended design, lower RMS values were recorded in the Anycubic (0.108 ± 0.010 mm) and eSUN (0.106 ± 0.008 mm) groups, while the highest was observed in the Elegoo group (0.135 ± 0.016 mm) (F(3, 42.2) = 19.946, *p* < 0.001). In the Connector design, RMS values ranged from 0.094 ± 0.006 mm (Anycubic) to 0.121 ± 0.005 mm (Elegoo), with statistically significant differences detected across groups (F(3, 43.8) = 98.414, *p* < 0.001).

**Figure 8:**
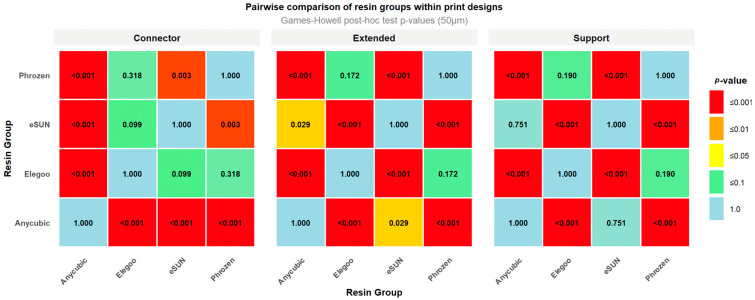
Heatmap of pairwise post hoc comparisons among resin groups (50 µm layer thickness).

Games–Howell post hoc test revealed significant differences among resin groups across the tested layer thicknesses. At 50 µm, in the Support design, Phrozen exhibited higher RMS values compared with eSUN (0.020 mm) and Anycubic (0.018 mm), while significant differences were also observed between Elegoo and eSUN (0.018 mm) and between Anycubic and Elegoo (0.015 mm). In the Extended design, Phrozen showed higher RMS values than Anycubic (0.021 mm) and eSUN (0.015 mm), and the Anycubic–Elegoo comparison (0.016 mm) was also significant. For the Connector design, significant differences were observed between Phrozen and Anycubic (0.014 mm), Anycubic and Elegoo (0.011 mm), Anycubic and eSUN (0.008 mm), and eSUN and Phrozen (0.006 mm), whereas the differences between Elegoo and eSUN and between Elegoo and Phrozen were not significant (Figure 8).

**Figure 9:**
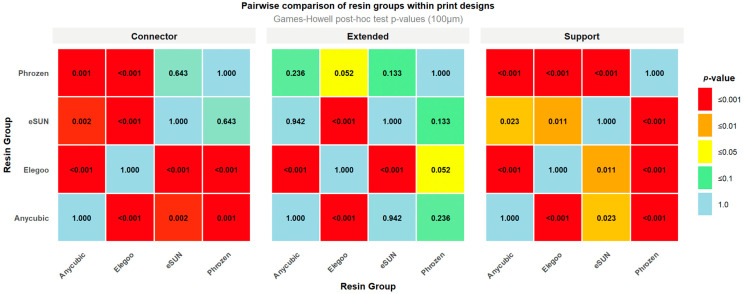
Heatmap of pairwise post hoc comparisons among resin groups (100 µm layer thickness).

At 100 µm, in the Support design, Phrozen exhibited lower RMS values compared with Elegoo (0.030 mm), eSUN (0.024 mm), and Anycubic (0.018 mm), while significant differences were also observed between Anycubic and Elegoo (0.012 mm) and between Elegoo and eSUN (0.018 mm). In the Extended design, marked differences were found between Elegoo and eSUN (0.029 mm) and between Anycubic and Elegoo (0.027 mm), while the comparison between Elegoo and Phrozen (0.016 mm) was borderline significant. For the Connector design, significant differences were detected between Anycubic and Elegoo (0.028 mm), eSUN and Elegoo (0.021 mm), Phrozen and Elegoo (0.018 mm), Anycubic and eSUN (0.007 mm), and Anycubic and Phrozen (0.010 mm), whereas the difference between eSUN and Phrozen was not significant (Figure 9).

### 3.2. Print Design-Based Comparison

**Figure 10:**
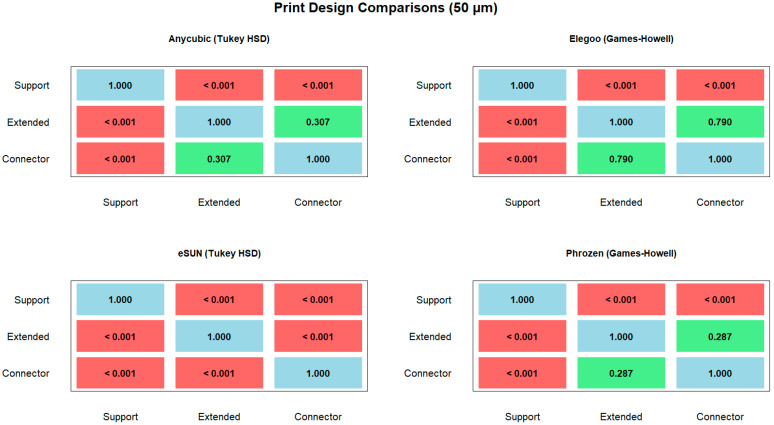
Pairwise comparisons of print designs (support, extended, connector) at 50 µm layer thickness for different resins. Post hoc tests: Tukey HSD (Anycubic, Elegoo, eSUN) and Games–Howell (Phrozen).

Pairwise post hoc comparisons revealed that at 50 µm layer thickness, significant differences were observed across most resin–design combinations, with Anycubic, Elegoo, and eSUN showing highly significant differences between Support vs. Extended and Support vs. Connector (*p* < 0.001, Tukey HSD), while Connector–Extended differences were generally not significant, except in eSUN (Table 2). For Phrozen, Extended–Support and Connector–Support were highly significant (*p* < 0.001, Games–Howell), whereas Connector–Extended showed no difference (Figure 10).

**Figure 11:**
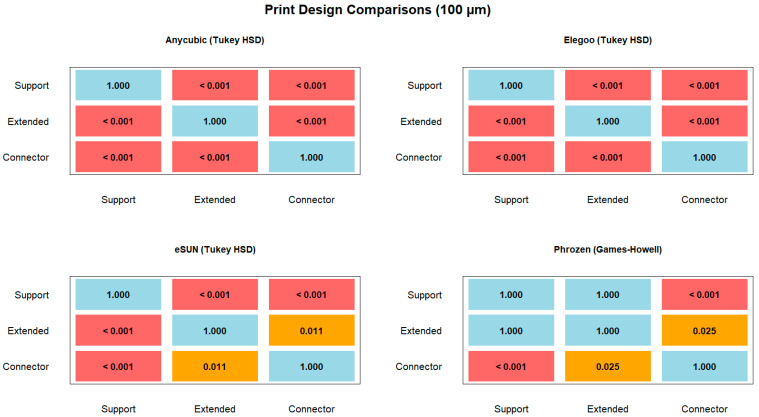
Pairwise comparisons of print designs (support, extended, connector) at 100 µm layer thickness for different resins. Post hoc tests: Tukey HSD (Anycubic, Elegoo, eSUN) and Games–Howell (Phrozen).

At 100 µm layer thickness, highly significant differences were detected for all pairwise comparisons in Anycubic, Elegoo, and eSUN (*p* < 0.001, Tukey HSD), except for the Connector–Extended contrast in eSUN, which was marginally significant (*p* = 0.011). In Phrozen, Extended–Support was not significant (*p* = 1.000, Games–Howell), but Connector–Support and Connector–Extended comparisons were significant (*p* < 0.05, Games–Howell) (Figure 11).

### 3.3. Layer-Based Comparison

**Figure 12:**
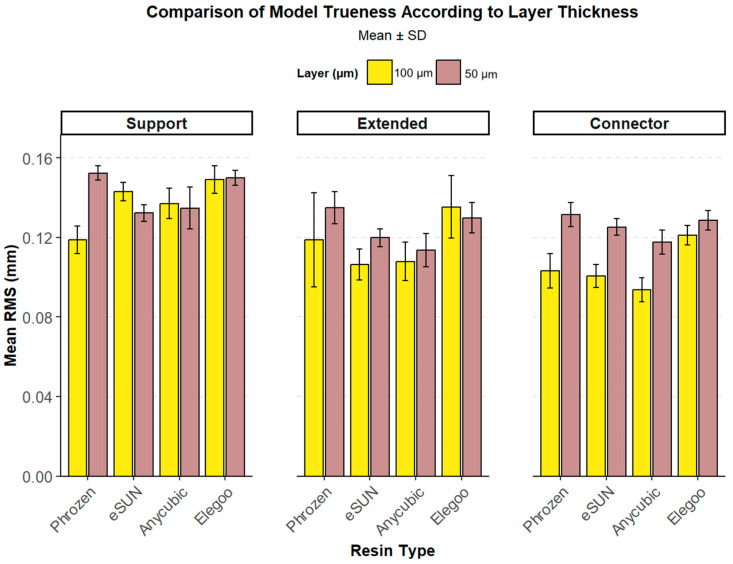
Comparison of model trueness (mean RMS ± SD) among resins at two-layer thicknesses (50 µm and 100 µm) across three print designs (Support, Extended, Connector). Yellow bars represent 100 µm and purple bars represent 50 µm layer thickness.

**Figure 13:**
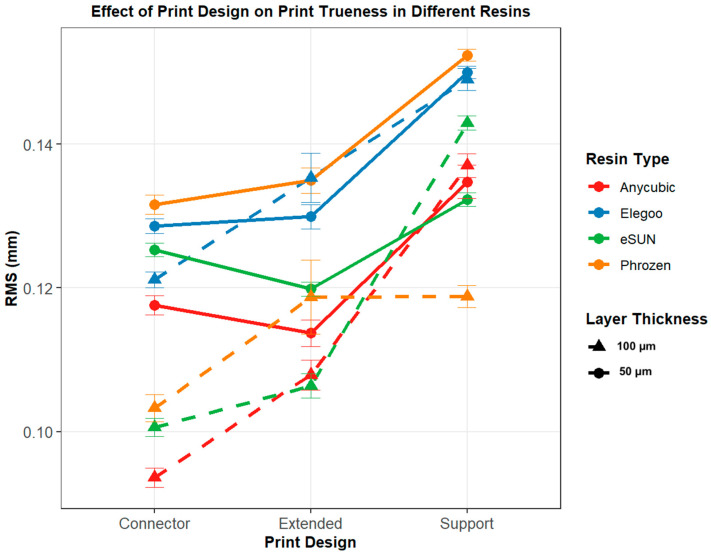
Trueness (RMS, mm) of 3D-printed models according to print design (connector, extended, support), resin type, and layer thickness. Solid lines represent 100 µm and dashed lines represent 50 µm. Error bars indicate standard error (SE).

Layer thickness affected trueness differently across resins and print designs. In Anycubic and Elegoo, significant differences were observed only in the connector design (*p* < 0.001), while eSUN and Phrozen showed consistently lower RMS values at 50 µm across all designs (all *p* < 0.05). These results suggest that the impact of layer thickness is resin- and design-dependent, with eSUN and Phrozen particularly benefitting from thinner (50 µm) printing (Table 4, Figure 12 and Figure 13).

### 3.4. Evaluation of Precision with Respect to Resins

**Figure 14:**
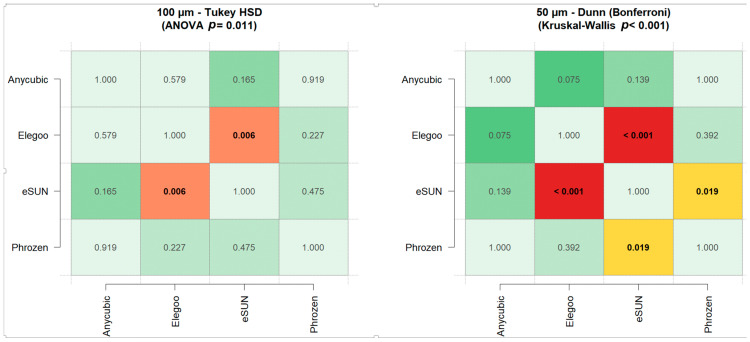
Pairwise post hoc comparisons of precision among resin groups at 100 µm (Tukey HSD, **left**) and 50 µm (Dunn–Bonferroni, **right**) layer thicknesses. Values represent pairwise *p*-values. Color scale indicates significance levels: green = not significant (*p* > 0.05), yellow = significant (*p* < 0.05), orange = very significant (*p* < 0.01), red = highly significant (*p* < 0.001).

Precision analysis demonstrated that resin type significantly influenced repeatability at both layer thicknesses. At 50 µm, the Kruskal–Wallis test indicated significant differences among the resins (χ^2^(3) = 18.597, *p* < 0.001, η^2^ = 0.135). Post hoc analysis revealed that eSUN exhibited significantly higher precision (lower RMS) compared with Elegoo (*p* < 0.001, Cohen’s d = 0.42) and Phrozen (*p* = 0.019, d = 0.40), while differences involving Anycubic were not significant (Figure 14, Table 5). At 100 µm, ANOVA showed a significant difference among the resins (F(3, 116) = 3.904, *p* = 0.011, η^2^ = 0.092). Post hoc comparisons confirmed that eSUN had higher precision than Elegoo (*p* = 0.006, Cohen’s d = 0.86), whereas no other pairwise differences reached significance (Figure 14, Table 5 and Table 6) [35].

## 4. Discussion

The rapid advancement of 3D printing technology has led to its increasingly widespread application in different branches of dentistry [10,27,36]. Compared to conventional techniques, 3D-printed models offer significant advantages in terms of accuracy, reliability, and efficiency in applications such as occlusal splints and surgical guides [20]. In orthodontics, 3D printing is now frequently utilized, particularly for the fabrication of surgical guides for miniscrew placement, custom orthodontic appliances, bracket transfer trays, and clear aligners [11,12,13]. Three-Dimensional models are also widely used for orthodontic study models, patient communication, and dental education [37]. Compared to traditional stone models, 3D-printed dental models can accommodate more complex geometries, offer greater dimensional precision, and exhibit improved structural durability [27,38].

Several critical considerations accompany the widespread use of 3D printing in orthodontics, particularly in aligner therapy. Inaccuracies during the manufacturing process may hinder the precise transfer of planned tooth movements such as rotations, intrusions, or anchorage control into the oral environment, leading to undesired movements or failure of the intended correction. Incomplete seating of aligners can further cause gingival impingement and patient discomfort. Moreover, such cumulative inaccuracies may necessitate an increased number of aligners, additional intraoral scans after appliance misfit, and ultimately reduce the predictability of treatment outcomes. Therefore, it is essential to analyze and control the factors that affect print accuracy to ensure reliable and clinically safe results.

Numerous factors have been reported to influence 3D printing accuracy [18,20]. One of the most critical factors is the type of 3D printer used. Comparative studies have evaluated printers utilizing different technologies such as SLA (stereolithography), DLP (digital light processing), and LCD (liquid crystal display) [4,18,19]. However, there is a lack of sufficient studies assessing the accuracy of printed models with different resins. Moreover, the limited existing studies have not directly evaluated maxillary or mandibular models [27]. In this context, our study represents the first to address this gap.

In studies evaluating printing accuracy, an RMS deviation of 0.25 mm is widely accepted as the clinical threshold for trueness [4,31,39,40,41]. This is based on the orthodontic literature, which states that the maximum planned tooth movement per aligner typically ranges between 0.25 and 0.30 mm [4,42,43]. Therefore, it is essential that models intended for generating orthodontic force via clear aligners exhibit errors below this limit. In our study, all resins, print designs, and layer thicknesses demonstrated results below this threshold.

The orientation of an object within the slicer software significantly influences its dimensional accuracy (trueness) and overall printing precision [16,44]. Objects can be oriented horizontally, vertically, or at an inclined angle, each option presenting specific advantages and limitations. A horizontal orientation shortens printing time by requiring fewer layers, whereas a vertical orientation allows multiple models to be arranged on the build platform simultaneously, which is advantageous for high-volume production [30,45,46,47]. In this study, vertical orientation was chosen as it enabled the fabrication of a greater number of models in a single printing run, a strategy particularly beneficial for in-house clear aligner production.

Another factor that influences the accuracy of 3D-printed dental models is the base design. While horseshoe-shaped models are advantageous for clear aligner fabrication, they are prone to deformation if not adequately supported. Camardella et al. [48] reported that models with either a flat base or a horseshoe design reinforced by a posterior bar exhibited greater dimensional stability compared to unsupported horseshoe designs. However, full-base designs such as the American Board of Orthodontists (ABO) standard lead to unnecessary resin consumption. To address this limitation, the present study investigated three alternative horseshoe configurations: models printed with conventional support structures, models reinforced with a posterior connector, and models with an extended base, aiming to improve stability while minimizing material use. The rationale for employing connectors was to limit potential widening of the model during the building process due to the increased weight of the structure. However, our results did not demonstrate a clear difference between the extended and connector designs. Nevertheless, both approaches contributed positively to printing accuracy. Special attention is required in retroclined upper and lower models with vertical orientations, where improper bonding between incrementally polymerized layers may occur at certain angles. In such cases, a slight inclination of the printing angle in the vertical axis may enhance layer integration and overall accuracy.

Although several factors affecting 3D printing accuracy have been examined, the impact of support usage and resin type on dimensional accuracy remains insufficiently explored. The aim of this study was therefore to compare orthodontic models fabricated from different resins, with and without support structures, using the same digital model under standardized conditions.

Support structures are often added to enhance adhesion to the build platform and prevent detachment during printing movements [49]. They also help reduce deformation and are typically placed in non-critical regions to avoid interfering with anatomical details [50,51,52]. For instance, occlusal and adjacent soft tissue surfaces can usually be printed without direct supports, and in many cases models can be printed flat on the build platform, reducing both printing time and material consumption [53].

Our findings indicate that additional supports are unnecessary when the posterior part of the model is extended (extended design) or when a connector is incorporated in the posterior region. These strategies provide sufficient stability and avoid disadvantages associated with supports. The higher deviations observed in support-based models may be attributed to surface marks left after support removal or distortions of the supports themselves, which can propagate as cumulative errors. While increasing the thickness or density of supports may mitigate these issues, extended and connector designs proved to be more rational and clinically practical alternatives.

In one study, it was reported that models printed with supports generally demonstrated higher accuracy [54]. However, the model used in our study was a mandibular model with teeth and therefore more complex, whereas that study did not employ a dental model. Moreover, parameters such as the resins and printers used, as well as the printing orientation of the models, differed from those in our study. Zhu et al. [23] evaluated the effects of different 3D printing angles (0°, 45°, and 90°) and the presence or absence of support structures on the dimensional accuracy of bracket transfer models and the precision of bracket placement. The highest accuracy was achieved in models printed at 90° with supports, whereas the lowest accuracy and greatest deviation were observed in models printed at 0° without supports. Models printed at 0°, particularly without supports, exhibited the largest errors in the vertical (Z) axis, with deviations around 0.285 mm.

In their in vitro study (2024), Namano et al. [33] investigated the effects of reducing support structures on the trueness and precision of complete maxillary dentures. They concluded that reducing the support structures in the palatal and border regions could preserve clinically acceptable fit while improving material efficiency and surface integrity [33].

In another in vitro study (2025), Namano et al. [55], evaluated the effects of different support structure designs and various post-polymerization procedures on the accuracy of 3D-printed complete dentures. Measurements were performed using a scanner with an accuracy of 10 µm and a repeatability of 4 µm. The obtained data were superimposed, and trueness and precision values were calculated using the RMS method. The researchers reported that when a tree-like support structure was used and the denture was placed at 60 °C for 30 min after polymerization, the dimensional accuracy of the tissue-contacting surface improved. This finding indicates that both the design of support structures and the post-polymerization protocol together influence the accuracy of 3D-printed complete dentures [55].

In the present study, two orthodontic resins (Elegoo and eSUN), one dental model resin (Phrozen, water washable), and one general-purpose resin (Anycubic Craftman) were selected to represent different categories of materials used in clinical and laboratory practice. The inclusion of water-washable resin was intended to emphasize its practical and economic advantages, as it can be cleaned with water rather than isopropyl alcohol. Since alcohol has several disadvantages, including odor, rapid vaporization, and ocular irritation, as well as higher costs, water-based post-processing offers a budget-friendly and safer alternative for routine in-office or laboratory workflows. The general-purpose resin, although not specifically developed for dental use, was chosen to evaluate whether lower-cost materials such as Anycubic could provide accuracy comparable to that of specialized dental or orthodontic resins, thereby supporting the feasibility of cost-effective digital orthodontic workflows.

When comparing resin performance, the orthodontic model resin eSUN and the Anycubic Craftsman resin exhibited the best outcomes, showing lower RMS values. In contrast, the Elegoo Orthodontic Resin demonstrated the poorest accuracy, while the Phrozen Dental water-washable resin underperformed in some designs and layer thicknesses. Specifically, we noted that resin formulations differ in polymer network density, filler content, and degree of conversion after photo-curing. These material properties directly influence dimensional stability and shrinkage, which in turn affect RMS deviations. The resin that demonstrated superior accuracy likely benefited from a higher cross-link density and reduced polymerization shrinkage, leading to better shape accuracy and lower deviation compared to other resins. It should also be noted that these outcomes may be partly specific to the printer used and the combined effects of the applied print settings. Although efforts were made to identify optimal curing intervals, overexposure can lead to thickening of the printed model, whereas insufficient curing may result in thinning and dimensional inaccuracies.

A recent study by Ling et al. [27] using a professional-grade printer with costly proprietary resins such as Formlabs reported lower shrinkage and slightly higher accuracy. According to their findings, nine different commercially available model resins were compared in terms of dimensional accuracy, volumetric shrinkage, and stability of accuracy over time, and all resins demonstrated clinically acceptable accuracy with errors remaining below 50 µm. However, the printer employed had a relatively small build platform and belonged to the professional-grade category, differing from the larger and more cost-effective desktop printer used in our study, which makes direct comparison inappropriate. Similarly, Pauls and Hornberg [41] investigated two professional-grade dental resins and two soy-based biodegradable resins using a professional laboratory printer and found no significant differences in accuracy between the materials. Their evaluation, however, relied only on manual caliper and digital linear software measurements.

Another important factor influencing printing accuracy is layer thickness, which has the potential to affect material properties [30,31,32]. Theoretically, in a printing system capable of optimally managing hardware and resin interactions, thinner layers would be expected to enhance printing accuracy. However, the reported influence of layer thickness on print accuracy has been inconsistent in the literature [30,31,39]. In contrast to this expectation and to some previous findings, our study demonstrated that a 100 µm layer thickness produced superior accuracy, particularly in vertically oriented models with greater height. This result may be explained by the accumulation of errors across the larger number of layers required in 50 µm printing, underscoring the critical role of Z axis precision. Moreover, peel forces between the release film and build platform increase proportionally with the number of layers, potentially compromising accuracy at thinner settings. The hardness level of the light-cured material during separation from the release film may also influence its structural integrity, while the penetration of light through the selected layer thickness is another important factor. In essence, this phenomenon may be related to the extent to which light can uniformly penetrate the material, as well as the volumetric changes that occur during the transition from liquid to solid. Beyond these aspects, additional parameters such as build platform motion, exposure times, release film properties, LCD resolution, resin temperature, resin viscosity, and polymer composition are also likely to influence printing accuracy, highlighting the multifactorial complexity of achieving optimal trueness in orthodontic model fabrication.

Layer heights of 50 µm and 100 µm, which are commonly applied in dental model printing, were selected. In many previous studies, root mean square (RMS) values have been used to assess dimensional deviations [4,39,56]. While RMS provides an overall quantitative measure, it does not indicate the specific location of deviations. Therefore, in this study, point-to-surface distance calculations were additionally visualized through color-coded maps, allowing identification of the regions where deviations were most pronounced (Figure 2, Figure 3, Figure 4 and Figure 5).

In the 3D printing process, exposure time is one of the most critical parameters, as it determines the duration each layer is subjected to UV light and directly influences print quality [57,58]. This parameter affects key factors such as detail resolution, dimensional accuracy, mechanical properties, and surface characteristics. Overexposure results in excessive polymerization, which can cause the loss of fine details, rounded edges, and overall enlargement of the model [58,59]. In such cases, negative features such as holes and gaps appear smaller than intended. Overexposed structures are typically harder and more brittle and may exhibit deformations such as warping or bending due to internal stresses [58]. In contrast, underexposure leads to insufficient polymerization [58]. This condition compromises the reproduction of fine details, causes dimensional shrinkage, and weakens structural integrity. Although underexposed models may appear softer and more flexible, they are mechanically fragile. Common defects include layer separation, detachment from the build platform, residual sticky resin, and glossy surface patches [57,58].

Therefore, identifying the optimal exposure time is essential to preserve fine details while ensuring sufficient mechanical strength [57,60]. In this study, eight reference models were fabricated for each resin type using the standard Exposure Range Finder (R_E_R_F) calibration file provided with the Anycubic Photon M7 Pro to establish the optimal curing time. A potential limitation, however, is that the exact optimal exposure intervals may not have been fully captured during this calibration process.

Additionally, fabrication parameters such as layer thickness, light settings (intensity and duration), build plate orientation, and mechanical factors (lift distance, lift and retract speeds, and light-off delay) may have influenced polymerization kinetics and dimensional accuracy. Despite standardization efforts, uncontrolled environmental factors, including ambient and resin temperature during printing, also represent inherent limitations. Moreover, since all 3D-printed resin models were re-scanned one week after fabrication, dimensional changes due to ongoing polymerization shrinkage over time should also be considered. Another limitation is that only a low-cost LCD-based printer was employed, and more expensive resins were not included, which may restrict the generalizability of the findings.

Future studies should therefore aim to systematically investigate the effects of printer-specific settings, resin temperature stabilization, and alternative calibration strategies to better define optimal printing and curing conditions. Comparative analyses across different 3D printing technologies and resin formulations, including both high-cost proprietary and affordable alternatives under standardized protocols, will be essential to establish more generalizable recommendations for improving dimensional accuracy, reproducibility, and clinical applicability of 3D-printed dental models

## 5. Conclusions

All resins and printing designs tested in this study demonstrated dimensional accuracy within orthodontically acceptable limits. Nevertheless, significant differences were observed depending on resin type, print design, and layer thickness. Printing at 100 µm generally provided superior accuracy compared to 50 µm, particularly in vertically oriented models with greater height, likely due to reduced error accumulation and minimized peel forces. Moreover, the shorter printing time associated with 100 µm layer thickness offers a practical advantage for in-office orthodontic model fabrication, enabling faster production workflows without compromising dimensional reliability. Extended and connector base designs outperformed support-based printing, suggesting that alternative stabilization strategies can enhance accuracy while reducing surface artifacts. Among the resins, eSUN Ortho Model Resin and Anycubic Craftsman Resin exhibited the most favorable results, whereas Elegoo Orthodontic Resin and Phrozen Water-Washable Dental Model Resin showed higher deviations under certain conditions, with Anycubic Craftsman Resin further emerging as a cost-effective option for orthodontic model production by combining clinically acceptable accuracy with reduced material costs. These findings emphasize that printing accuracy is governed by multifactorial interactions, including resin composition, build design, and printing parameters. Clinically, the results support the use of optimized resin–design–layer thickness combinations to achieve reliable, precise, and time-efficient in-office orthodontic model fabrication for aligner production.

## Figures and Tables

**Table 1:** Resins and print design types used in the study.

Resin Category	Resin Name and Type	Manufacturer (Location)	Print Orientation	Print Design Types (Support, Extended, Connector)
Orthodontic Resin	Elegoo Orthodontic Resin	Elegoo Inc. (Shenzhen, China)	Vertical	N = 21/design
Orthodontic Resin	eSUN Orthodontic Resin	Shenzhen Esun Industrial Co., Ltd. (Shenzhen, China)	Vertical	N = 21/design
Dental Model Resin	Phrozen Water-Washable Dental Model Resin	Phrozen Tech Co., Ltd. (Hsinchu City, Taiwan)	Vertical	N = 21/design
General Purpose Resin	Anycubic DLP Craftsman Resin (Beige)	Anycubic (Shenzhen, China)	Vertical	N = 21/design

Note: Each resin was printed at two layer thicknesses (50 µm and 100 µm), resulting in 126 models per resin and 504 models in total.

**Table 2:** Comparison of the accuracy of different resins in various print designs at 50 µm.

Design	Resin Type	Mean ± SD	Min–Max	*p*-Value
Support	Anycubic	0.135 ± 0.011	0.113–0.152	<0.001 ***
	Elegoo	0.150 ± 0.004	0.143–0.158	
	eSUN	0.132 ± 0.004	0.126–0.139	
	Phrozen	0.152 ± 0.004	0.145–0.161	
Extended	Anycubic	0.114 ± 0.008	0.097–0.125	<0.001 ***
	Elegoo	0.130 ± 0.008	0.115–0.144	
	eSUN	0.120 ± 0.004	0.114–0.127	
	Phrozen	0.135 ± 0.008	0.122–0.149	
Connector	Anycubic	0.118 ± 0.006	0.109–0.130	<0.001 ***
	Elegoo	0.129 ± 0.005	0.121–0.137	
	eSUN	0.125 ± 0.004	0.119–0.133	
	Phrozen	0.132 ± 0.006	0.123–0.143	

Values are presented as mean ± SD (standard deviation) and min–max ranges. Significant differences are indicated by *p*-values (*** = *p* < 0.001). Welch ANOVA test.

**Table 3:** Comparison of the Accuracy of Different Resins in Various Print Designs at 100 µm.

Design	Resin Group	Mean ± SD	Min–Max	*p*-Value
Support	Anycubic	0.137 ± 0.008	0.122–0.148	<0.001 ***
	Elegoo	0.149 ± 0.007	0.136–0.158	
	eSUN	0.143 ± 0.005	0.136–0.153	
	Phrozen	0.119 ± 0.007	0.104–0.130	
Extended	Anycubic	0.108 ± 0.010	0.089–0.125	<0.001 ***
	Elegoo	0.135 ± 0.016	0.113–0.159	
	eSUN	0.106 ± 0.008	0.092–0.119	
	Phrozen	0.119 ± 0.024	0.080–0.157	
Connector	Anycubic	0.094 ± 0.006	0.082–0.103	<0.001 ***
	Elegoo	0.121 ± 0.005	0.112–0.128	
	eSUN	0.101 ± 0.006	0.092–0.111	
	Phrozen	0.103 ± 0.009	0.089–0.118	

Values are presented as mean ± SD (standard deviation) and min–max ranges. Significant differences are indicated by *p*-values (*** = *p* < 0.001). Welch ANOVA test.

**Table 4:** Pairwise comparison of RMS values between 50 and 100 µm layer thickness within each resin and design.

Resin	Design	Test Used	*p*-Value (Sig.)
Anycubic	Support	Paired *t*-test	0.4902 (ns)
Anycubic	Extended	Paired *t*-test	0.0586 (ns)
Anycubic	Connector	Paired *t*-test	<0.001 (***)
Elegoo	Support	Paired *t*-test	0.5798 (ns)
Elegoo	Extended	Paired *t*-test	0.1595 (ns)
Elegoo	Connector	Wilcoxon signed-rank	<0.001 (***)
eSUN	Support	Paired *t*-test	<0.001 (***)
eSUN	Extended	Paired *t*-test	<0.001 (***)
eSUN	Connector	Paired *t*-test	<0.001 (***)
Phrozen	Support	Paired *t*-test	<0.001 (***)
Phrozen	Extended	Wilcoxon signed-rank	0.0105 (*)
Phrozen	Connector	Paired *t*-test	<0.001 (***)

Significant differences are indicated by *p*-values (*** = *p* < 0.001, * = *p* < 0.05, ns = not significant).

**Table 5:** Descriptive statistics of precision measurements at 50 µm.

Resin Group	N	Mean	SD	Median	IQR
Anycubic	30	0.074	0.113	0.050	0.019
Elegoo	30	0.086	0.132	0.061	0.021
eSUN	30	0.047	0.012	0.043	0.018
Phrozen	30	0.073	0.091	0.052	0.025

Min: Minumum, Max: Maximum, SD: Standard deviation.

**Table 6:** Descriptive statistics of precision measurements at 100 µm.

Resin Group	N	Mean	SD	Min	Max
Anycubic	30	0.055	0.011	0.032	0.086
Elegoo	30	0.059	0.013	0.036	0.093
eSUN	30	0.049	0.010	0.034	0.077
Phrozen	30	0.053	0.013	0.031	0.079

Min: Minumum, Max: Maximum, SD: Standard deviation.

## Data Availability

The original contributions presented in the study are included in the article, further inquiries can be directed to the corresponding author.
